# Interoperability Framework of the European Health Data Space for the Secondary Use of Data: Interactive European Interoperability Framework–Based Standards Compliance Toolkit for AI-Driven Projects

**DOI:** 10.2196/69813

**Published:** 2025-04-23

**Authors:** Rada Hussein, Amelie Gyrard, Somayeh Abedian, Philip Gribbon, Sara Alabart Martínez

**Affiliations:** 1 Ludwig Boltzmann Institute for Digital Health and Prevention Salzburg Austria; 2 Trialog Paris France; 3 Fraunhofer Institute for Translational Medicine and Pharmacology Frankfurt am Main Germany; 4 Fraunhofer Cluster of Excellence for Immune-Mediated Diseases (CIMD) Frankfurt Germany; 5 TIC Salut Social Foundation Barcelona Spain

**Keywords:** artificial intelligence, European Health Data Space, European interoperability framework, healthcare standards interoperability, secondary use of health data

## Abstract

The successful implementation of the European Health Data Space (EHDS) for the secondary use of data (known as EHDS2) hinges on overcoming significant challenges, including the proper implementation of interoperability standards, harmonization of diverse national approaches to data governance, and the integration of rapidly evolving AI technologies. This work addresses these challenges by developing an interactive toolkit that leverages insights from 7 leading cancer research projects (Integration of Heterogeneous Data and Evidence towards Regulatory and HTA Acceptance [IDERHA], European Federation for Cancer Images [EUCAIM], Artificial intelligence Supporting Cancer Patients across Europe [ASCAPE], Personalised Health Monitoring and Decision Support Based On Artificial Intelligence and Holistic Health Records [iHelp], Central repository for digital pathology [Bigpicture], Piloting an infrastructure for the secondary use of health data [HealthData@EU] pilot, and improving cancer diagnosis and prediction with AI and big data [INCISIVE]) to guide in shaping the EHDS2 interoperability framework. Building upon the foundations laid by the Towards the European Health Data Space (TEHDAS) joint action (JA) and the new European Interoperability Framework (EIF), the toolkit incorporates several key innovative features. First, it provides interactive and user-friendly entry modules to support European projects in creating their own interoperability frameworks aligned with the evolving EHDS2 requirements technical and governance requirements. Second, it guides projects in navigating the complex landscape of health data standards, emphasizing the need for a balanced approach to implementing the EHDS2 recommended standards for data discoverability and sharing. Third, the toolkit fosters collaboration and knowledge sharing among projects by enabling them to share their experiences and best practices in implementing standards and addressing interoperability challenges. Finally, the toolkit recognizes the dynamic nature of the EHDS2 and the evolving regulatory landscape, including the impact of AI regulations and related standards. This allows for continuous adaptation and improvement, ensuring the toolkit remains relevant and useful for future projects. In collaboration with HSbooster.eu, the toolkit will be disseminated to a wider audience of projects and experts, facilitating broader feedback and continuous improvement. This collaborative approach will foster harmonized standards implementation across projects that ultimately contribute to the development of a common EHDS2 interoperability framework.

## Introduction

During the past years, the European Commission (EC) has aimed to unlock the potential of health data for providing better health care services and advancing research and innovation while upholding ethical and privacy principles. In 2020, the European strategy for data [1] proposed to establish common European data spaces to govern access and reuse of data from various sectors across the European Union (EU), starting with the European Health Data Space (EHDS) [2] as the first common data space across Europe. The EHDS will mainly empower individuals to take control of their health data across Europe (primary use of data, known as EHDS1 [European Health Data Space for the primary use of data]), in addition to reusing the health data in research, innovation regulatory activities, and policy-making (secondary use of data, known as EHDS2 [European Health Data Space for the secondary use of data]) [3].

The EHDS regulation was proposed on May 3, 2022, adopted by the European Parliament on April 24, 2024 [4], and is expected to enter into force by Autumn 2024. The EHDS2 obligations will be applied around 2028, except for some data categories, such as clinical trial data and human genetic data, that are expected to be implemented by 2030.

Meanwhile, the EC is establishing the main infrastructure of the EHDS, namely MyHealth@EU (for EHDS1), based on the lessons learned from the epSOS project cross-border health care services, and building on the xShare initiative for data portability), and HealthData@EU pilot (for EHDS2) [2,3]. Moreover, the Towards the European Health Data Space (TEHDAS) Joint Action (JA) [5] was launched to prepare the ground for the harmonized implementation of EHDS2 in the EU member states by developing the guidelines and technical specifications for smooth access to health data.

**Figure 1:**
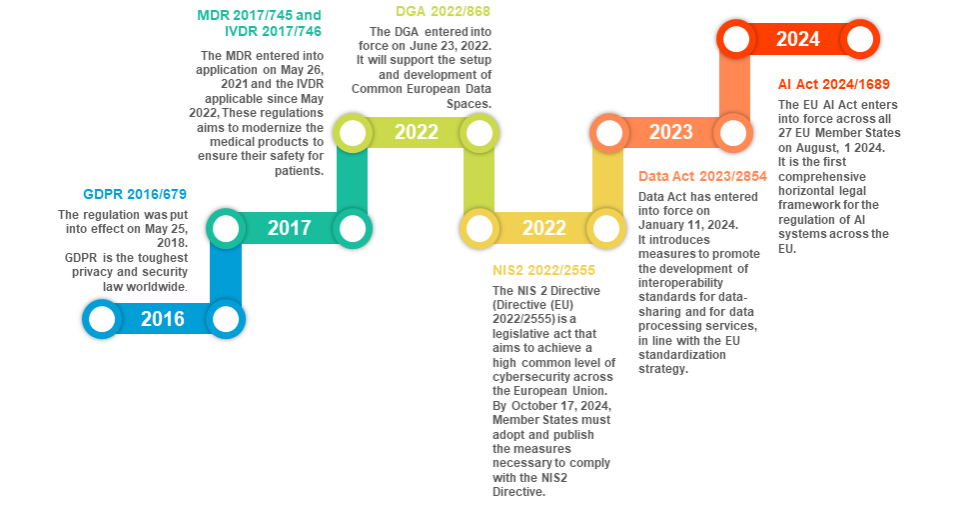
Timeline of the European Union data regulations and frameworks shaping the European Health Data Space Regulation.

The EHDS governance is built on the EU legal frameworks (Figure 1), mainly, the General Data Protection Regulation (GDPR), the Data Governance Act (DGA), the Medical Devices Regulation (MDR), In Vitro Diagnostics Regulation (IVDR), the Data Act, and the Network & Information Security (NIS2) Directive [2,6,7], in addition to the recently adopted AI (artificial intelligence) Act that shapes the European approach to building trustworthy AI [8]. The EHDS complements these regulations and will provide additional tailor-made rules when needed.

Recently, the HealthData@EU Pilot identified the common elements for health data access and data use within the legal frameworks of the participating nodes [9], namely the Biobanking and BioMolecular resources Research Infrastructure (BBMRI; European Research Infrastructure), Health Data Lab (Germany), Danish Health Data Authority (Denmark), Findata (Finland), Health Data Hub (France), Sciensano (Belgium), Norwegian Directorate of eHealth (Norway), and Croatian Institute of Public Health (Croatia). This work aimed to harmonize legal and ethical data access procedures, security requirements, and GDPR citizen access rights compliance for cross-border use of data. The performed analysis reflected that nodes vary to a great extent regarding the scope of available data, the legal basis for making the data available and accessible, and responding to data misuse. There are also major differences in the degree of centralization of the decision on data access.

To harmonize the EHDS implementation across the member states, the EHDS established a new independent advisory and regulatory body, called the EHDS Board, that facilitates the exchange of information and cooperation among member states and with the EC [6], such as: coordinating the practices among Health Data Access Bodies (HDABs) in member states, exchanging best practices with the EC to support the legislation, and sharing information on EHDS2 risks and incidents.

The EC rolling plan for AI standardization (2024) highlighted the ongoing policy, legislation, ethical, and technical standardization activities [10]. The European Committee for Electrotechnical Standardization (CEN-CENELEC) created a Joint Technical Committee (JTC 21) to address AI standardization in Europe, applying both a bottom-up approach (similar to International Organization for Standardization (ISO)/International Electrotechnical Commission (IEC) JTC 1 Subcommittees (SC) 42 “ISO/IEC JTC 1/SC 42 Artificial Intelligence”), and a top-down approach concentrating on a long-term plan for European standardization and future AI regulation. Notably, the SC 42 published standards [11] on (1) big data overview, vocabulary, reference architecture, and (2) AI covering bias in AI systems and AI-aided decision making, overview of trustworthiness in AI, assessment of the robustness of neural networks, use cases, overview of computational approaches for AI systems. The different workgroups of the SC 42 are still developing the standards on (1) foundational AI standards, (2) big data ecosystem, and (3) AI trustworthiness.

The EC also established the AI watch as a hub for the Joint Research Centre’s scientific research on AI [12]. In 2023, the AI Watch published a report on the state of the art of AI's current and near-future applications in medicine, health care, and well-being [13]. At the implementation level, the AI for Health Imaging (AI4HI) [14] project network was launched to develop cancer imaging data repositories and AI solutions based on medical imaging to improve clinical practice. The involved projects are PRIMAGE, CHAIMELEON, EuCanImage, INCISIVE, ProCAncer-I, and RadioVal.

**Figure 2:**
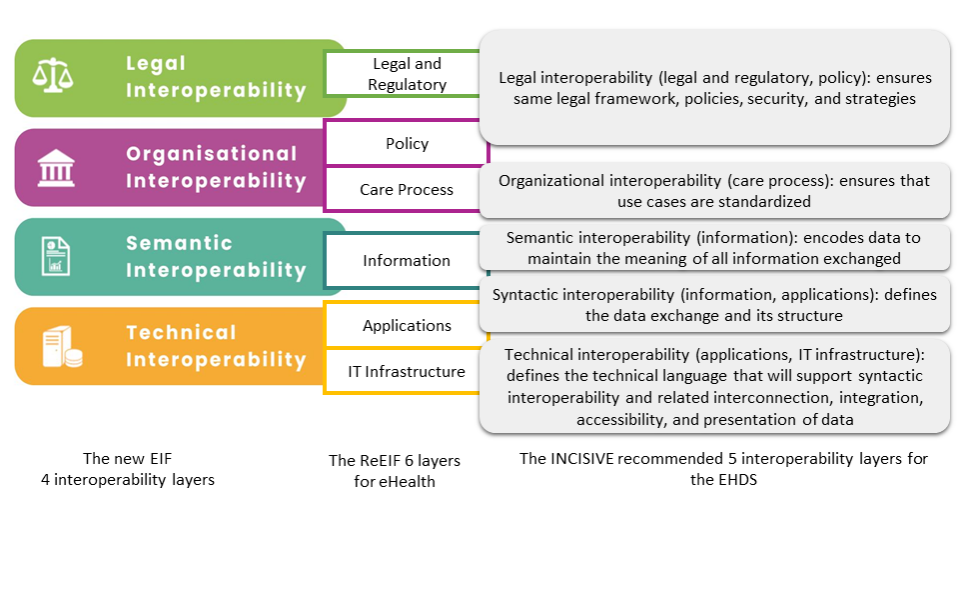
The interoperability layers of the European Interoperability Framework, Refinement of the eHealth European Interoperability Framework, and the INCISIVE project. EHDS: European Health Data Space; EIF: European Interoperability Framework; ReEIF: Refinement of the eHealth European Interoperability Framework.

On the other hand, the EC adopted the new European Interoperability Framework (EIF) in 2017 [15] to connect public administrations, businesses, and citizens across Europe [16]. The EIF conceptual model comprises 4 levels of interoperability namely, legal, organizational, semantic, and technical interoperability, associated with the underlying 12 principles and 47 recommendations for proper implementation. The eHealth Network refined the EIF for the eHealth domain into 6 layers in the Refinement of the eHealth European Interoperability Framework (ReEIF) [17]. In 2023, the INCISIVE project [18] provided its interoperability framework based on the ReEIF as recommended input for the anticipated interoperability framework for the EHDS [19]. Figure 2 depicts the 4 layers of the EIF and its interlinking with the ReEIF 6 layers and the recommended 5 overlapping layers by the INCISIVE project.

## Standards and Interoperability in the European AI-Driven Projects

Interoperability is the cornerstone of digital medicine, especially in the AI era, which necessitates the availability of various high-quality datasets [20]. The lack of proper interoperability implementation hinders AI implementation in the real world, as highlighted by the recent study published by the Scientific Foresight Unit of the European Parliamentary Research Service in 2022 [21]. At the industry level, MedTech Europe and COCIR partnered to present a medical technology industry perspective on the standards, levels of interoperability, standards, profiles, and specifications relevant to digital health [22]. Furthermore, recent studies addressed the need for an interoperability framework for the EHDS that uses the EIF for managing specifications for interoperability [23-27] while considering the other aspects of data security, privacy, governance, and quality. The proposed interoperability framework should also be based on approved international interoperability standards for semantics and syntax, for example, the standards described and examined in [28-30].

In response to this challenge, we have created a synergy between AI-data projects with cancer use cases based [31] in October 2023, through the HSbooster.eu initiative for fostering standards in EU-funded projects. These current projects shape the EHDS implementation, for example, the Integration of Heterogeneous Data and Evidence towards Regulatory and HTA Acceptance (IDERHA), European Federation for Cancer Images (EUCAIM), HealthData@EU pilot, and other projects [31]. We started by creating a template for the EHDS2 interoperability framework based on the EIF. Then, we mapped the used standards in 6 projects with the created template [32]. We compared the health-standardized models, ontologies, and terminologies used in these projects and mapped their relevance to EHDS2 [32]. We also examined the AI tools, techniques, and standards, as well as the federated data infrastructure in these projects.

In this work, we created an EHDS2 interoperability framework based on the EIF. We used the intensive expertise and lessons learned from the synergy projects to identify the underlying interoperability standards and best practices in implementation. These standards cover health standards and Internet of Medical Things (IoMT) semantic interoperability [33] in the context of interoperability standards for health data, knowledge graphs–related technologies, Smart Applications REFerence ontology (SAREF), Data quality framework (DQF), Patient-Generated Health Data (PGHD), AI reasoning, federated approaches, security and privacy. This toolkit aims to support existing and future projects in creating their interoperability framework in compliance with EHDS2 governance and technical requirements.

## Existing Interoperability Framework Tools and Guides

We investigated the interoperability tools and guides provided by EU-funded projects and international organizations during the past few years. Table 1 summarizes the key findings and compares these tools in terms of their compliance with the EIF and EHDS2 requirements [23].

The listed tools in Table 1 provide guidance on creating interoperability toolkits in general, while the TEHDAS and INCISIVE projects focus on EHDS2. The IHE integration profiles indicate how to define use cases in any project of clinical data interoperability and promote the coordinated use of established standards such as Digital Imaging and Communications in Medicine (DICOM) and Health Level 7 (HL7). Both IHE and eHAction tools mainly target EHDS1. All tools do not provide concrete guidance on how to select the proper standards for each EIF layer relevant to the EHDS2 recommended standards, for example, standards for data discoverability (meta-data standards), and other related aspects, including data quality, IoMT, PGHD, and others.

**Table 1:** Main recent guides and tools of the interoperability frameworks.

Project/International organization	Tool/guide (year)	Scope	EIF^a^ compliance	EHDS2^b^ compliance
World Bank Group’s ID4D^c^ Initiative [34]	ID4D Practitioner’s Guide (2019)	To help practitioners design and implement identification systems that are inclusive and trusted—in accordance with the 10 Principles on Identification for Sustainable Development and other international standards and good practices	Based on the EIF 4 layers	Not specifically for the health domain
eHAction JA^d^ [35]	Interoperability Guide (2020)	To support health care providers in planning and procuring standards-based interoperable solutions	Based on the ReEIF^e^	Mainly for EHDS1^f^
TEHDAS JA^g^ [28]	EHDS Semantic interoperability framework (2022)	Providing EHDS2 data life-cycle and recommendations to enhance interoperability within the HealthData@EU	Based on the EIF	Target EHDS2
INCISIVE^h^ project [19,36]	Recommendations of interoperability standards (2023) INCISIVE HL7^i^ FHIR^j^ Implementation Guide (2024)	To contribute to the EUCAIM^k^ interoperability framework, as input for the EHDS interoperability framework	Based on the ReEIF	For projects managing clinical data and medical images
WHO^l^ [37,38]	Ethics and governance of AI for health (2021 and 2024)	Recommendations for consideration by governments, technology companies, and health care providers to ensure the appropriate use of AI in health	Related to the legal interoperability layer of the EIF	Necessary for EHDS2
IHE^m^ [39]	IHE profiles for the exchange of documents in European eHDSI^n^	IHE defines Integration Profiles that use existing standards for system integration, providing effective interoperability and efficient workflow	Related to the organizational/technical interoperability layers of the EIF	Mainly EHDS1

^a^EIF: European Interoperability Framework.

^b^EHDS2: European Health Data Space for the secondary use of data.

^c^ID4D: Identification for Development.

^d^eHAction JA: eHealth Action.

^e^ReEIF: Refined eHealth European Interoperability Framework.

^f^EHDS1: European Health Data Space for the primary use of data.

^g^TEHDAS JA: Towards the European Health Data Space Joint Action.

^h^INCISIVE: improving cancer diagnosis and prediction with AI and big data.

^i^HL7: Health Level 7.

^j^FHIR: Fast Healthcare Interoperability Resources.

^k^EUCAIM: European Federation for Cancer Images.

^l^WHO: World Health Organization.

^m^IHE: Integrating the Healthcare Enterprise.

^n^eHDSI: eHealth Digital Services Infrastructure.

In addition, the recent guides of the World Health Organization (WHO) on regulatory considerations on AI for health [38], and the ethics and governance of artificial intelligence for health [37] can shape the legal, regulatory, and policy layer accompanying the adoption of the AI Act.

## EHDS2 Interoperability Framework Toolkit

**Figure 3:**
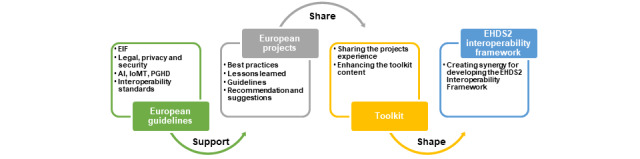
The European Health Data Space for the secondary use of data interoperability framework: concept and process of creation. AI: artificial intelligence; EHDS2: European Health Data Space for the secondary use of data; IoMT: Internet of Medical Things; PGHD: patient-generated health data.

The main concept of creating the toolkit is to incorporate the European guidelines, interoperability standards, and best practices of European projects in real-world implementation for shaping the EHDS2 interoperability framework (Figure 3).

**Figure 4:**
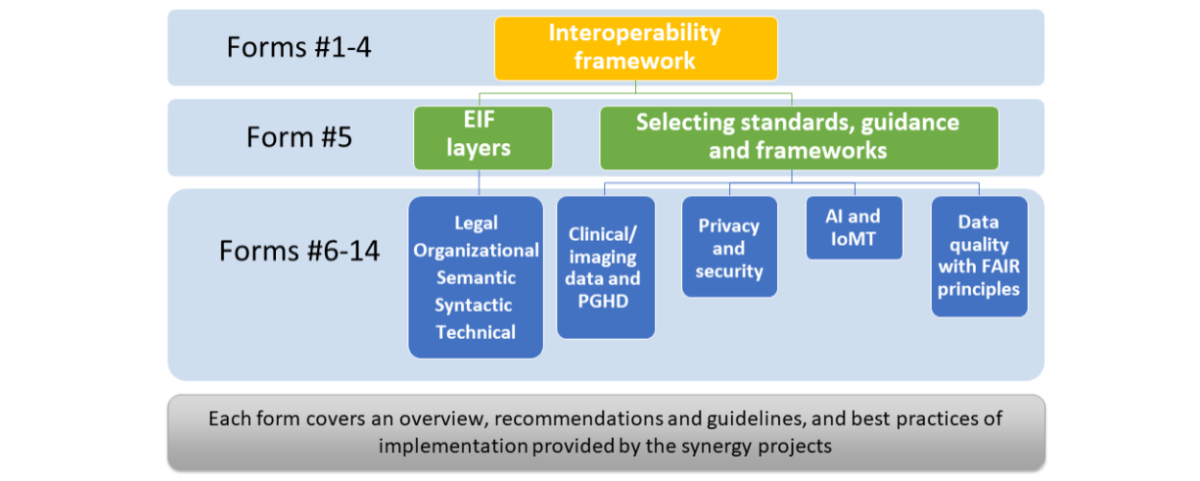
The toolkit of European Health Data Space for the secondary use of data interoperability framework: main components and sections. EIF: European Interoperability Framework; FAIR: Findability, Accessibility, Interoperability, and Reusability; IoMT: Internet of Medical Things; PGHD: patient-generated health data.

Thus, the toolkit aims to provide the existing and future EU projects with an interactive and user-friendly entry interface designing, writing, and printing the interoperability framework in compliance with EHDS2. It facilitates the selection of the standards for each EIF layer and provides more guidance on the proper implementation of these standards by providing best practices from other projects or international organizations (Figure 4).

## Standards and Guidelines Selection Module

To design the toolkit standards and guideline modules, we first created a template for mapping the interoperability standards between TEHDAS, INCISIVE project, and the created list of implemented standards used in the HSbooster.eu synergy projects (Table 2). We did not intend to compare the standards implemented in these projects but rather highlight how these projects fulfill the interoperability goals and share their learned lessons. Thus, Table 2 highlights the most important standards implemented in these projects, without meaning that the other projects do not implement the proper standards.

We adopted the TEHDAS recommended categories of EHDS2 interoperability standards, as follows:

1. Data discoverability: the World Wide Web Consortium (W3C) Data Catalog Vocabulary (DCAT)-Application Profile (AP), as well as the anticipated extension of DCAT-AP for health (HealthDCAT-AP) being developed by the HealthData@EU pilot.
2. Enabling semantics interoperability: the Observational Medical Outcomes Partnership (OMOP)-Common Data Model (CDM)- developed by the Observational Health Data Sciences and Informatics (OHDSI).
3. Health data exchange (DICOM for imaging data and the HL7 Fast Healthcare Interoperability Resources (FHIR) for health records).

Then, we identified the related ISO standards for data quality, PGHD, AI reasoning, federated approaches, security, and privacy.

The template was refined by adding the INCISIVE standardization suggestions [19], on the following:

- Use cases and health care process definition
- Data exchange without losing the semantic meaning with terminologies
- Clinical documents exchange
- Medical image exchange
- Clinical data and events exchange
- Common data model to index or group data
- Standardize the database with archetypes to present data in the user interface.

**Table 2:** Summary of mapping the interoperability standards to the European Health Data Space for the secondary use of data template based on the European Interoperability Framework using the Towards the European Health Data Space and Improving Cancer Diagnosis and Prediction with AI [artificial intelligence] and Big Data Results.

EIF^a^	TEHDAS^b^ [6] Governance model of the EHDS2^c^ of the data life-cycle	INCISIVE^d^ [19] Recommendations of interoperability standards (managing clinical data and medical images)	HSbooster.eu synergy projects [31,32] (the main health and IoMT^e^ standards^f^)
Legal	The EHDS^g^ will build on the legal frameworks laid out by the EU^h^ regulations (Figure 1)	Legal and security standards and FAIRi principles. The detailed explanation is provided in INCISIVE’s Deliverable 7.3 Data donation legal framework ISOj 14971 Medical Devices Risk Management Assessment IECk 62304 Medical device software - Software life cycle processes	ISO/IEC 27001:2022- Information security, cybersecurity, and privacy protection IEEEl 7000 Series- AI ethical considerations ISO/IEC 42001_2023 – AI – Management system and requirements
Organizational	Adapting the national policies in compliance with the EHDS regulations Harmonizing the EU member states health policies and the delivery of health services and medical care	The IHE^m^ integration profiles identified the care process, actors, transactions, and events	IHE integration profiles ISO/IEC 5259-1:2024 Artificial intelligence — Data quality for analytics and machine learning ISO 9000: Data Quality Management Systems standards Data quality frameworks Data FAIRification framework
Semantic	Standards on data discoverability: (DCAT-APn) Standards that allow semantic interoperability: (OMOP-CDMo)	SNOMED CTp (Clinical data) LOINCq (Lab data) OHDSIr vocabularies	SNOMED, LOINC, ICDs, RxNorm, etc ISO 13606-Health Informatics- Electronic Health Record —Parts 1,2,4 (Reference model/ Archetype interchange specification / Security) ISO 11073-10101 and ISO 11073-10201- Health informatics — Device Interoperability (Point-of-care medical device communication — Domain information model/Nomenclature) ISO 23903:2021 Health informatics — Interoperability and integration reference architecture — Model and framework Standards on data discoverability: HealthDCAT-AP OMOPt for enabling data analysis ISO/IEC 22989: AI concept and terminology
Syntactic	Standards that facilitate communication: FHIRu (Clinical data) DICOMv (Imaging data)	FHIR (All data) DICOM (Imaging data)	HL7, FHIR (Clinical data) DICOM (Imaging data) ISO/IEEE 11073-20601 and ISO/IEEE 11073-20701 (PGHD- Device Interoperability and optimized exchange protocol) EN/ISO 13606: Health Informatics- Electronic Health Record —Parts 3,5(Reference archetypes and term lists/Interface specification)
Technical	Federated peer-to-peer (P2P) network Secure processing environment	JSON or XML	Semantic web languages such as W3Cw RDFx, RDFSy, OWLz, SPARQLaa, JSON-LDab, etc JSON or XML (medical data) ISO accreditation for the EHDS2 secure processing environment, for example, ISO/IEC 27001 IEEE 3652.1, Guide for Architectural Framework and Application of Federated Machine Learning

^a^EIF: European Interoperability Framework.

^b^TEHDAS JA: Towards the European Health Data Space Joint Action.

^c^EHDS2: European Health Data Space for the secondary use of data.

^d^INCISIVE: improving cancer diagnosis and prediction with AI and big data.

^e^IoMT: Internet of Medical Things.

^f^The complete list of standards is available in the toolkit. The table highlights the main standards used in health and IoMT

^g^EHDS: European Health Data Space.

^h^EU: European Union.

^i^FAIR: Findability, Accessibility, Interoperability, and Reusability.

^j^ISO: International Standards Organization.

^k^IEC: Internation Electrochemical Commission.

^l^IEEE: Institute of Electrical and Electronics Engineers.

^m^IHE: Integrating the Healthcare Enterprise.

^n^DCAT-AP: Data Catalogue vocabulary Application Profile for data portals.

^o^OMOP-CDM: Observational Medical Outcomes Partnership Common Data Model.

^p^SNOMED CT: Systematized Nomenclature of Medicine – Clinical Terms.

^q^LOINC: Logical Observation Identifiers Names and Codes.

^r^OHDSI: Observational Health Data Sciences and Informatics.

^s^ICD: International Classification of Diseases.

^t^OMOP mapping (linking the semantic layer with the syntactic layer)

^u^FHIR mapping (linking the semantic layer with the syntactic layer).

^v^DICOM: Digital Imaging and Communications in Medicine.

^w^W3C: World Wide Web Consortium.

^x^RDF: Resource Description Framework.

^y^RDFS: Resource Description Framework Schema.

^z^OWL: Web Ontology Language.

^aa^SPARQL: SPARQL Protocol and RDF Query Language.

^ab^JSON-LD: JSON for Linked Data.

Deciding between the HL7 FHIR and OMOP CDM standards can be complex, as each offers distinct advantages to different needs within health care systems. HL7 FHIR is particularly advantageous due to its flexibility, allowing for personalized profiles, extensions, and the use of local code systems. It enables the exchange of granular clinical data through its resource-based framework and RESTful API. This adaptability, combined with its support for incremental adoption, makes FHIR highly suitable for organizations looking to integrate real-time data exchange into their new or existing infrastructure. Additionally, its alignment with established standards like HL7 v2 and v3 reinforces its utility in a wide range of health care applications.

Conversely, OMOP CDM shines in facilitating research collaborations, data analysis, and evidence generation through its standardized and harmonized approach to observational health care data. Designed primarily to support observational research, OMOP uses OHDSI vocabularies (for this reason, OMOP is often situated as a semantic layer standard) to semantically code clinical terms instead of using CodeSystems as FHIR does, but both use the same international terminologies. While OMOP is particularly powerful for research purposes and large-scale data consumption, it may be less suitable as a messaging standard, as its implementation often requires extensions to cover all relevant health care domains. As such, it is more appropriate for systems focused on data analytics rather than operational data exchange.

In determining which standard to adopt, the decision depends on the intended use case. HL7 FHIR is better suited for systems that prioritize flexible, real-time clinical data exchange and customization through extensions and local adaptations. In contrast, OMOP is ideal for those focused on research, standardized data analysis, and evidence generation across multiple health care organizations. Integrating both standards can offer significant benefits, promoting interoperability between clinical and research environments, and leveraging FHIR’s strengths in real-time data sharing and OMOP’s robust framework for data analysis and evidence generation. Fortunately, achieving interoperability between FHIR and OMOP is being carried out by the HL7 and OHDSI communities. For example, the developed semantic Web-based FHIR-Ontop-OMOP system demonstrates great potential in health care AI applications enabled by the interoperability of FHIR and OMOP CDM [40] and the FHIR to OMOP FHIR Implementation Guide [41]. The relevant FHIR and OMOP mapping resources and implementation guide are also listed in the syntactic standards form of the toolkit to provide a proper linking of the semantic layer with the syntactic layer.

## Interoperability Framework Entry Module

We also used INCISIVE recommended methodology for the design and implementation of the interoperability framework [19], in providing the entry modules of the main components of the main interoperability framework sections: (1) needs analysis, (2) selection of data and information, (3) processes and use cases, (4) interoperability standards and implementation guides, (5) risk analysis, (6) maintenance plan, (7) specification, design and planning of interoperability phases, (8) recommendations and future actions, and (9) implementation guide publication.

**Figure 5:**
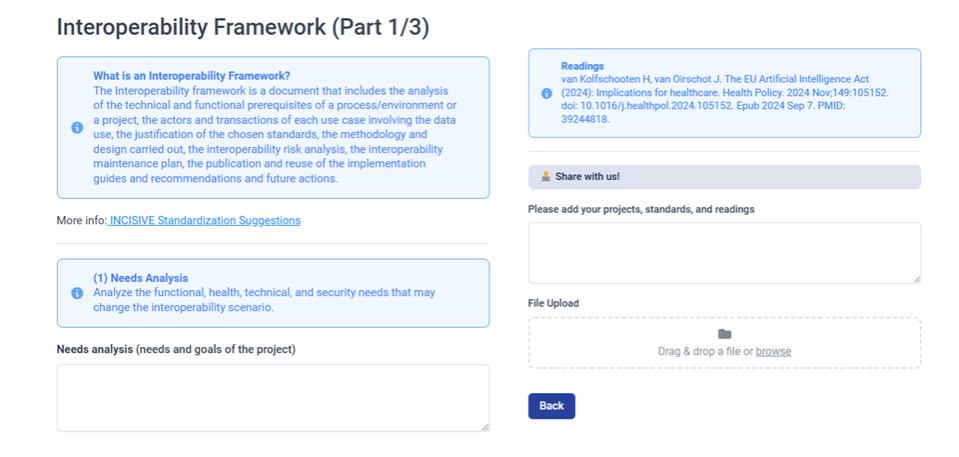
The interoperability toolkit: a snapshot of the entry module of the interoperability framework and how the projects can share their lessons learned.

**Figure 6:**
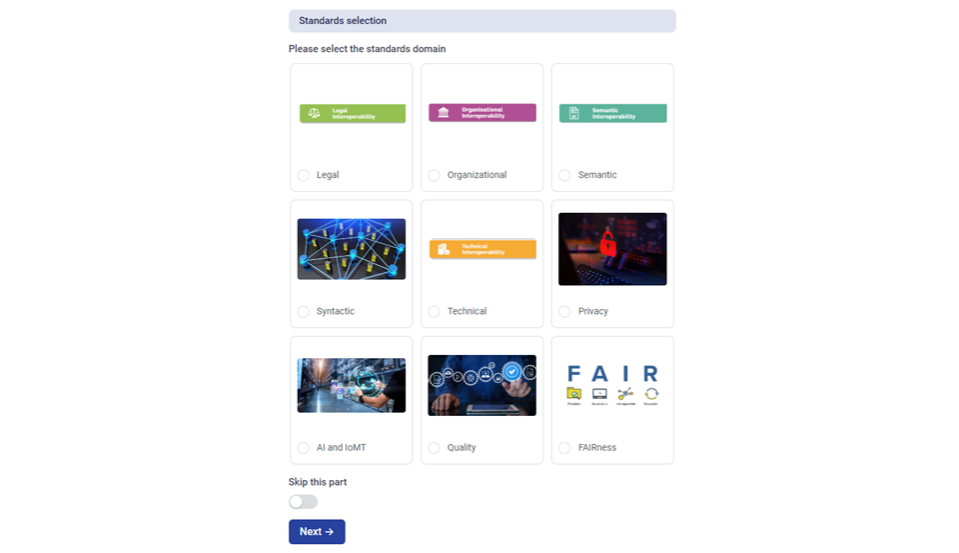
The interoperability toolkit: a snapshot of the module of the standards selection.AI: artificial intelligence; IoMT: Internet of Medical Things.

**Figure 7:**
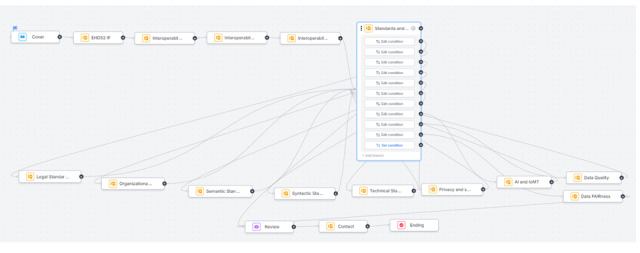
The logic for navigating the interoperability toolkit.

Finally, we used the Fillout platform [42] in creating the interactive interface of the toolkit (Figures 5-7). The Integration of Heterogeneous Data and Evidence towards Regulatory and HTA Acceptance (IDERHA) project in collaboration with the HSbooster.eu created the first version of the toolkit [43]. This is a part of IDERHA’s plan to align with the EHDS2 standards and interoperability requirements [44].

## Identifying the AI Risks and Legal Obligations According to the EU AI Act Using the Toolkit

**Figure 8:**
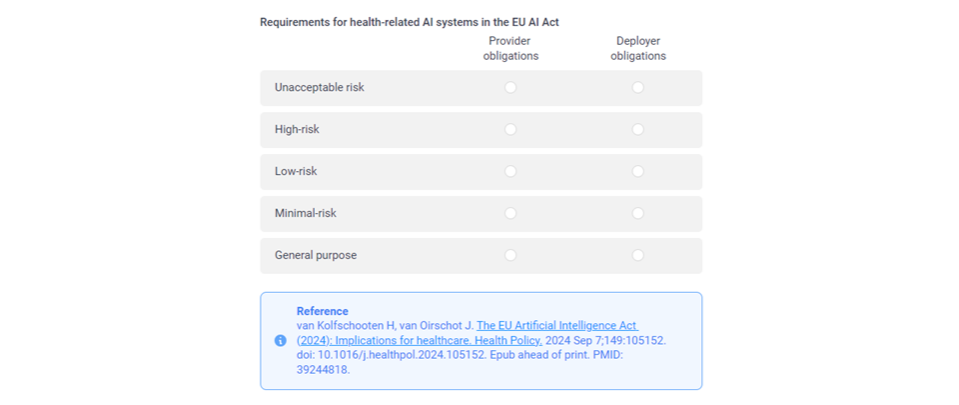
Identifying the associated artificial intelligence risk and obligations by referring to the guidance. AI: artificial intelligence; EU: European Union.

The AI Act applies to all sectors and it does not identify certain rules for the health care domain. Recently, a study was published to highlight the implications of the AI Act for the health care sector and give a comprehensive overview of the new legal obligations for various health care stakeholders (technical developers, health care professionals, and public health authorities) [45]. We adopted this study in the toolkit as a guideline for identifying the AI risk in EU projects as a part of the risk analysis of the interoperability framework (Figure 8).

Now, the toolkit lists the standards and the lessons learned from synergy projects formed through the HSbooster.eu in cancer [31,32]. We also encourage other projects to share their expertise and used standards through the toolkit designated fields for input and uploading files. We enabled the Fillout settings for respondent notifications, including the link to edit submissions, to allow the respondents to revise their answers at any time. Furthermore, the automatic notification email to the respondents will contain a summary of the responses for creating the interoperability framework that can be saved or printed.

Ultimately, the toolkit can also be used at the international level for creating the EHDS2 interoperability frameworks with countries outside the European region, especially the notable attention as an inspiring model for transatlantic collaborative health data use and AI development [46].

## Conclusion

In preparation for a harmonized implementation of EHDS2, most of the funded European projects applying AI in health are planning for the proper alignment with the EHDS2 requirements in data governance and interoperability [36,44].

The described toolkit in this work will support the AI-driven project in creating the interoperability framework based on the EHDS2 governance and technical needs. We will share the toolkit with the health standards experts in TEHDAS JA and the HealthData@EU pilot to get their feedback on how to improve and maintain it. We also will share the proposed EHDS2 interoperability framework with IHE Europe to investigate the integration profiles for closing the gaps between EHDS1 and EHDS2. Moreover, we plan to conduct a workshop during the Medical Informatics Europe (MIE2025) to establish a dialogue with the health informatics community on how to use the toolkit as one of the inputs for the EHDS2 interoperability framework. We will also discuss the challenges of keeping the toolkit up to date with the evolving AI standards landscape through hosting the toolkit by the HSbooster. Finally, it will be essential to assess the impact of using the toolkit to ensure that it meets its goal, and delivers the expected benefits to the health data ecosystem. This will also be accompanied by conducting a thorough review of the EHDS2 interoperability achievements and challenges across the member states driven by the real-world implementation of the EHDS.

## Abbreviations

AI: artificial intelligence
AI4HI: AI for Health Imaging
ASCAPE: Artificial intelligence Supporting Cancer Patients across Europe
BBMRI: the Biobanking and BioMolecular resources Research Infrastructure
CDM: common data model
CEN-CENELEC: the European Committee for Electrotechnical Standardization
DCAT: data catalog vocabulary
DCAT-AP: DCAT Application profile for data portals in Europe
DGA: the Data Governance Act
DICOM: Digital Imaging and Communications in Medicine
DQF: Data Quality Framework
EC: European Commission
EHDS: European Health Data Space
EHDS1: European Health Data Space for the primary use of data
EHDS2: European Health Data Space for the secondary use of data
EIF: European Interoperability Framework
EU: European Union
EUCAIM: European Federation for Cancer Images
FHIR: fast healthcare interoperability resource
GDPR: general data protection regulation
HDABs: Health Data Access Bodies
HL7: Health Level 7
IDERHA: Integration of Heterogeneous Data and Evidence towards Regulatory and HTA Acceptance
IEC: International Electrotechnical Commission
iHelp: Personalised Health Monitoring And Decision Support Based On Artificial Intelligence And Holistic Health Records
IoMT: Internet of Medical Things
ISO: international organization for standardization
IVDR: In Vitro Diagnostics Regulation
JA: joint action
JTC 21: Joint Technical Committee
MDR: Medical Devices Regulation
NIS2: the Network and Information Security Directive 2
OHDSI: observational health data sciences and informatics
OMOP: observational medical outcomes partnership
PGHD: patient-generated health data
ReEIF: Refinement of the eHealth European Interoperability Framework
SAREF: Smart Applications REFerence ontology
TEHDAS: Toward the European Health Data Space
W3C: World Wide Web Consortium
WHO: World Health Organization
